# Gene expression profiling in peripheral blood lymphocytes for major depression: preliminary cues from Chinese discordant sib-pair study

**DOI:** 10.1038/s41398-021-01665-4

**Published:** 2021-10-19

**Authors:** Chan Wu, Danfeng Wang, Kangkang Niu, Qili Feng, Hanwei Chen, Haibing Zhu, Hui Xiang

**Affiliations:** 1 South China Normal University-Panyu Central Hospital Joint Laboratory of Basic and Translational Medical Research, Guangzhou Panyu Central Hospital, Guangzhou, China; 2 Guangdong Provincial Key Laboratory of Insect Developmental Biology and Applied Technology, Guangzhou Key Laboratory of Insect Development Regulation and Application Research, Institute of Insect Science and Technology, School of Life Sciences, South China Normal University, Guangzhou, 510631 China; 3 The Affiliated Brain Hospital of Guangzhou Medical University, Guangzhou, China; 4 Department of Psychiatry, Guangzhou Panyu Central Hospital, Guangzhou, China

**Keywords:** Clinical genetics, Comparative genomics, Predictive markers, Depression

## Abstract

The etiology of major depressive disorder (MDD) involves many factors such as heredity and environment. There are very few MDD-related studies in Chinese population using twin or sib-pairs for depression-control samples. Here we used the microarray approach and compared gene expression profiling of peripheral blood lymphocytes from 6 sib-pairs discordant on lifetime history of MDD. Within sib-pair differentially expressed genes are obvious fewer in the 1st, 2nd, and 5th compared with those in the 3rd, 4th, and 6th sib-pairs. Gene expression pattern of these DEGs distinguished MDD individuals from the normal one in 3rd, 4th, and 6th sib-pair but not in the 1st, 2nd, and 5th pair, suggesting heterogeneity of different sib-pairs and somewhat commonalities among the 3rd, 4th, and 6th sib-pairs. Comprehensive protein interaction network analysis revealed two key genes *PTH* and *FGF2* in a dominant network where the majority of the genes were significantly down-regulated. *PTH* was significantly down-regulated in all the sib-pairs while *FGF2* was in the 3rd, 4th, and 6th sib-pairs. KEGG enrichment analysis of all the DEGs in networks showed that *PTH* and related genes were significantly enriched in the pathway of parathyroid hormone secretion, synthesis, and action while *FGF2* and related genes were significantly enriched in the pathways of cancer and specifically breast cancer. Generally reduced expression of these genes in peripheral blood lymphocytes of MDD individuals implied their functional repression associated with MDD. Pending validation in more samples, the findings in this study provided valuable cues for understanding the potential mechanism of MDD, as well as potential markers for the diagnosis and treatment of depression in the Chinese population.

## Introduction

Major depressive disorder (MDD) is a chronic mental disease with low mood, loss of interest, and lack of energy as the core symptoms. It is characterized by a high recurrence rate high suicide rate and high mortality rate. It is expected to be the major cause of the disease burden by 2030 [1]. Depression has become an important public health problem and seriously affects the quality of life of individuals and families [2]. In China, the lifetime prevalence of depression is 6.9% and the 12-month prevalence is 3.6% [3] and the number of people with depression is the highest in the world.

Depression is a complex disease that is associated with the central nervous system, hypothalamic-pituitary-Adrenal (HPA) axis, inflammation, metabolomics, immune system, and brain-derived neurotrophic factor [4, 5]. The etiology of depression also involves multiple genetic and environmental factors [6, 7]. Large sample genomic screening studies have been generated from those of European ancestry, Japanese and Chinese samples respectively, proposed nearly different sets of genetic risk loci [7–9], suggesting heterogeneity in genetics as well as environments of this disease. Specifically, GWAS with Chinese women with recurrent MDD identified two risk-related loci near the *SIRT1* gene and in an intron of the *LHPP* gene [8]. In addition to genomic deciphering of the mechanism of MDD, recent progresses on comparative transcriptomics provide new insights to elucidate the molecular mechanism of MDD and provide cues for identifying biomarkers and better treatment for depression [10–14]. For example, studies on brain comparative transcriptomics indicated altered glial, endothelial and ATPase activity related to MDD [11]; Sha and Banihashemi detected a cerise of pathways including immune response and transmembrane transport, which were associated with regional grey matter volume change regional structural variations in MDD [13], A single-nucleus transcriptomics of the prefrontal cortex in major depressive disorder found greatest dysregulation occurred in deep layer excitatory neurons and immature oligodendrocyte precursor cells [15]. In addition to the brain, gene expression profiling in blood was also reported to be affected by MDD [16–19], providing cues of inflammation in MDD and new insights in the molecular pathways involved.

It is notable that different studies may have inconsistent results, due to the complex biological and non-biological factors of depression, mainly involving differences in diagnostic criteria, specific dilemmas of genetic and imaging research methods. In addition, the depressed patients in many studies are random, with genetic heterogeneity among different individuals, resulting with potentially unavoidable noise interference. Interestingly, some surveys have found that depression tends to occur in one of the sib-pairs or even twins, while another may be normal for the long term (only 20% of male monozygotic twin pairs and 38% of female monozygotic twin pairs show consistency with the disease) [20]. Such sample models provide a unique opportunity to explore the pathogenesis of depression. For example, Zhu et al. conducted a genome-wide comprehensive analysis of DNA methylation and transcriptome in peripheral blood monocytes from 79 monozygotic twin pairs discordant on lifetime history of MDD and suggests epigenetic regulation may be associated with alteration of gene expression related to MDD [21]. In particular, several studies have used sib-pairs methods to explore the effects of family on psychopathological similarities and differences among siblings [22, 23]. Till now, almost all the researches using these unique materials are based on European and American population. Evidence from Asia samples is thus urgent, which may shed new light on the understanding pathogenesis of depression.

China has the highest number of MDD people but there was little research on the pathological mechanism of depression for Chinese sib-pairs, and almost most of the previous studies used a variety of xenogeneic cells in the postmortem brain tissue. Yang et al. examined the association of genetic polymorphisms with MDD susceptibility and treatment response using 181 Han Chinese with MDD and 80 healthy controls, which found the *CNR1* is a promising candidate for the genetic association study of MDD and understand how genetic polymorphisms are associated with the pathophysiology of major depressive disorder [24]. Another study explored the relationship between overweight and thyroid function in first-episode untreated Chinese patients with MDD at different ages of onset [25]. It is the same way that using low-coverage whole-genome sequencing of 5,303 Chinese women with recurrent MDD and 5,337 controls to identify possible loci contributing to the risk of MDD [8]. Lymphocytes are key monocyte immune cells involved in inflammation, and the immune system and inflammation are related to the pathological mechanisms of depression [26]. In this study, sampling 6 Chinese sib-pairs discordant of MDD (including 2 pairs of twins) as samples, we investigated gene expression profiling of peripheral blood lymphocytes using RNA microarray technology followed by statistical analysis, network analysis, and functional enrichment analysis, to preliminary explore the influence of MDD in blood. Our study provides the cues from the Chinese sib-pair samples, implying that repression of PTH and cancer-related pathways might be associated with MDD in these people.

## Materials and methods

### Subjects

We collected Chinese sib-pairs discordant of MDD (2 pairs of twins) from Guangzhou Panyu Central Hospital. The pairs were well matched in gender, age, et al. The study was approved by the Ethics Review Committee of the Guangzhou Panyu Central Hospital. All participants provided written informed consent prior to proceeding with the procedures related to the study. Their sibling were interviewed and they agreed to be included in our research program and were willing to provide blood samples.

### MDD diagnosis

All the patients with depression were diagnosed and evaluated by experienced psychiatrists through the DSM-IV-TR Axis I Disorders-Patient Edition (SCID-I/P) specific diagnostic tools, and DSM-IV depression was used as the diagnostic standard for diagnosis and clinical evaluation to determine lifetime and current depression. All eligible and interested subjects signed the Informed Consent Form before the trial. A discordant sib-pair is defined as one of the sib-pair that meets the criteria for a lifetime history of MDD, and his / her compatriot does not. Finally, 6 sib-pairs were selected in further analysis in this study (Table 1).

**Table 1:** Clinical characteristics of sib-pairs.

Sibling pair number	1	2	3	4	5	6
Gender^*^	M	O	M	F	M	F
Age of patient (year)	35	17	18	27	43	17
Age of normal (year)	35	12	23	26	43	21
Twins or not	yes	no	no	no	yes	no
Age difference	0	5	5	1	0	4
First onset age of patient (year)	35	16	16	24	43	15
Age stage of sibling pair	≥35	12–17	18–35	18–35	≥35	17–21
Suicide score	7	39	27	39	0	29
Total score of compulsion	0	7	0	8	3	13
Total score of anxiety	18	0	21	0	13	0
Mania score	0	4	4	0	0	0
Age of first visit (year)	35	17	17	27	43	15
First course of disease (month)	4	12	12	24	4	1
Hamilton Depression Scale (HAMD) of patients	31	23	25	25	20	10
Marital status (depression / normal)^#^	1/0	0/0	0/0	1/1	1/1	0/0
Working conditions (depression / normal)^&^	1/1	0/0	0/1	1/1	1/1	0/0

^*^M represents a male-male sibling pair, F represents a female-female sibling pair, and O represents a opposite-sex sibling pair.

^#^0 means unmarried and 1 means married.

^&^0 means not working and 1 means working.

### Inclusion / exclusion criteria

Only complete sib-pairs were eligible to participate in this study. Our samples belong to the type of sib-pair, so it was divided into inclusion/exclusion criteria for patients with depression and healthy compatriots. Inclusion criteria for patients with depression included: (1) meeting the diagnostic criteria for depression in the Diagnostic and Statistical Manual of Mental Disorders V (DSM-V); (2) aged between 18 and 45; (3) more than 6 years of education (primary school or above); (4) no history of electroconvulsive therapy within 3 months; (5) patients of first-episode and non-medication, the total score of HMDA ≥ 17; (6) understand the content of the study, hope to attend and be able to complete the whole experiment, and were willing to provide blood samples. The main exclusion conditions included: (1) had a history of neurological or major physical diseases; (2) had other psychiatric disorders (such as schizophrenia, obsessive-compulsive disorder, or phobia); and (3) has a history of alcohol and drug abuse or dependence. Among them, the inclusion criteria for the healthy control of sib-pairs were slightly different:(1) must be siblings (excluding parents or children); (2) not suffering from depression or other mental illness; (3) aged between 18 and 45; (4) more than 6 years of education (primary school or above); (5) understand the content of the study, hope to participate in and complete the whole experiment, and were willing to provide blood samples. The exclusion criteria were the same as those for patients with depression.

### Other measures

Each sib-pair was required to complete the case report form, including general sociodemographic information, medical history, family history, lifestyle, use of psychoactive substances, endocrine testing, and various scales. The severity of current depressive symptoms was assessed by the Hamilton Depression Scale (HAMD) and the Hamilton Anxiety Scale (HAMA). Reports of suicide thoughts were measured through suicide scales. Bipolar disorder was preliminarily excluded using the manic symptom scal (Bech- Rafaelsen). To investigate whether there were some genetic factors, genetic research family questionnaire was used to record the family history of sib-pair. Participants have tested the cortisol and adrenocorticotropic hormone to obtain endocrine test results.

### Isolation and preservation of lymphocytes

We took fresh anticoagulant 1 ml, mix with whole blood and tissue homogenate diluent (Cat#:2010C1119) at 1:1, and carefully added to the liquid surface of 2 ml cell separation solution, centrifuge at 1500–2000 rpm for 15 min (horizontal rotor with a radius of 15 cm). At this time, the liquid in the centrifuge tube was divided into four layers from top to bottom. The first layer was a plasma layer. The second layer was cyclic milky white lymphocytes. The third layer was a transparent separation liquid layer. The fourth layer was the erythrocyte layer. We collected the second layer of cells and put them in a test tube containing 4–5 ml of cell washing solution (Cat#: 2010 × 1118). After fully mixing, we centrifuged at 1500–2000 rpm for 10–30 min. The precipitation was washed twice to obtain the desired cells. Blood samples were processed and stored in a refrigerator at −80 °C for later use.

### Extraction, purification and quality control of RNA

Total RNA was extracted from lymphocytes using Trizol Reagent (Cat#15596-018, Life technologies, Carlsbad, CA, US) according to the standard operating procedures provided by the manufacturer. The extracted total RNA was qualified by Agilent Bioanalyzer 2100 (Agilent Technologies, Santa Clara, CA, US) electrophoresis and purified by RNeasy micro kit (Cat#74004, QIAGEN, GmBH, Germany) and RNase-Free DNase Set (Cat#79254, QIAGEN, GmBH, Germany). The concentration and quality of RNA were determined by NanoDrop ND-2000 spectrophotometer (biological analyzer). The qualified RNA could be used for subsequent microarray experiments.

### Microarray

The total RNA was sent to Shanghai Biotechnology Corporation for microarray establishment and raw data analysis. Briefly, RNA sample was amplified and labeled by Agilent expression microarray kit, Low Input Quick Amp Labeling Kit, One-Color (Cat.# 5190-2305 total RNA technologies, Santa Clara, CA, US) with standard operating procedure, and the labeled cRNA was purified with RNeasy mini kit (Cat.# 74106 and QIAGEN, GmBH, Germany). According to the standard hybrid process and Gene Expression Hybridization Kit (Cat.# 5188-5242 Magi Technologies, Santa Clara, CA, US) provided by Agilent expression microarray, the sample size of hybrid cRNA was 1.65 μg that was roll-hybrid in a rolling hybridization furnace Hybridization Oven (Cat.# G2545A, Agilent technologies, Santa Clara, CA, US) at 65 °C, 10 rpm for 17 h and washed in the staining dishes (Cat.# 121, Thermo Shandon, Waltham, MA, US). The reagent used for the washing sheet is Gene Expression Wash Buffer Kit (Cat.# 5188-5327, Agilent Technologies, Santa Clara, CA, US).

The chip that completes the hybridization was scanned by Agilent Microarray Scanner (Cat.# G2565CA and Agilent technologies, Santa Clara, CA, US), and the software was set to Dye channel: Green, Scan resolution = 5 μm, PMT 100%, 10%, 16 bit. Row data were filtered by Feature Extraction software 10.7 (Agilent Technologies, Santa Clara, CA, US), and finally normalized by the limma package in R software, using the algorithm of Quantile, to generate expression data of each gene. The probes that did not detect signals in all samples were removed from further analysis.

### Identification of differentially expressed genes (DEGs) potentially associated with MDD

The original data were normalized by the limma package in software R, and the output was an expression matrix file. We combined two approaches for identifying DEGs potential associated with MDD in each sib-pair. Firstly, within each pair, the candidate DEGs should have two-Fold changes in expression between the patient and the normal. Secondly, between 6 MDD patients and 6 related normal sibs, the candidate DEGs should have significantly different expression levels based on paired t-test.

### Upset plotting, principle components analysis (PCA) and hierarchical clustering of DEGs

DEGs from each sib-pair were then combined for Upset plotting at the online platform OmicStudio (https://www.omicstudio.cn/), principal component analysis (PCA) and hierarchical clustering at the online platform OmicShare Tools (https://www.omicshare.com/tools/).

### Protein interaction network analysis

Protein interaction network analysis of interested DEGs was generated by the STRING11.0 platform (https://string-db.org/). Genes that had no interaction with any other genes were removed from further analyses. The network was further processed using Cytoscape software 3.7.1.

### Functional enrichment analysis

Gene Ontology (GO) enrichment and KEGG enrichment analyses of the interesting gene sets were generated using the online platform OmicsBean (http://www.omicsbean.cn/).

## Results

### Sib-pairs

Table 1 showed the characteristics of the sib-pairs. Among the 6 sib-pairs, the 1st and 5th are wins. The age ranged from 12 to 43 and the age gap of each sib is not more than 5. Totally there are four male and 2 female patients. Of the six patients, one showed relatively lower HAMD (No.6, 10). Combined with other indexes such as Suicide score and total score of compulsion, she was diagnosed with MDD.

In terms of marital status and working conditions, except that the 1st pair had different marital status, the depressive patient of 3rd sib-pair had no job while his compatriot had job, there were no obvious differences between depressive patient and health control of each sib-pair. In addition, all six patients with depression were first-episode without medication, but the age of the first episode was different, three were adolescent, two were young, and the other one was more than 40 years old. It is notable that none of the six sib-pair had a family history of mental illness.

### DEGs potential associated with MDD

**Fig. 1:**
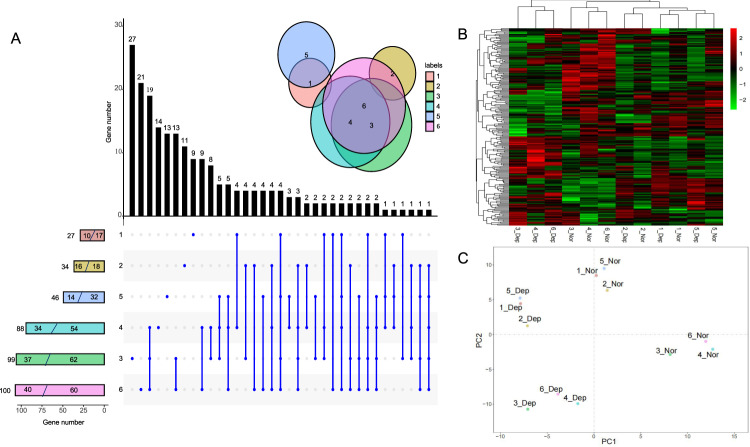
The overall pattern of gene expression in peripheral blood lymphocytes of the six sib-pairs. **A** Upset plot of differentially expressed genes of 6 sib-pairs (fold-change > 2 or < 0.5 and paired *T*-test < 0.05). The Gene number refers to the number of differentially expressed genes in each pair of sib-pairs. The first number in the bar is the number of up-regulated genes, and the second number is the number of down regulated genes. The blue dots and lines show the specific genes and common genes between sib-pairs, and the black bar chart shows their corresponding number. The Wayne diagram shows the intersection of the differentially expressed genes between them. **B** Heatmap of differentially expressed genes in 6 sib-pairs. It divides sibling pairs into two groups, 1st, 2nd, and 5th and 3rd, 4th and 6th. Among them, 1st, 2nd, and 5th sib-pairs are clustered according to sibling pairs, while 3rd, 4th and 6th sib-pairs are clustered according to normal and depression. **C** PCA plot of differentially expressed genes of 6 sib-pairs.

Considering genetic heterogeneity among the six sib-pairs and the small sampling size, we made efforts to identify potential DEGs in each of the sib-pair, by a uniform criterion taking into account of the fold change of expression level within each pair as well as a significant difference in expression level between the six patients and the relative sibs. Finally, the first, second, and fifth pairs had 27, 34, and 46 DEGs respectively, which is obviously lower than the third, fourth, and sixth pairs had (88, 99, and 100, respectively) (Fig. 1A). Upset Plotting indicated that a considerable proportion of the DEGs were unique to each sib-pair, while the 3rd, 4th and 6th pair shares quite a few common DEGs (Fig. 1A). The results of Wayne diagram analysis consistently indicate that the 3rd, 4th, and 6th pairs are largely overlapping whereas the 1st, 2nd, and 5th were somewhat scattered distributed, suggesting unique characteristics of these three pairs (Fig. 1A). Heatmap Hierarchical clustering of DEGs showed that the 3rd, 4th, and 6th sib-pairs could significantly distinguish between depression and normal individuals, while for 1st, 2nd, and 5th, compared with depression and normal type, they clustered by sib-pair, indicating that the gene expression similarity of sib-pair was higher. When we performed PCA analysis (Fig. 1C), we still found that 3rd, 4th, and 6th sib-pairs were separated from depression and normal on the principal component axis, suggesting that there was a great difference between them. It is interesting that although clustering did not separate depression from normal for the 1st, 2nd, and 5th sib-pairs, PCA analysis did. There may be some commonalities in depression individuals in 1st, 2nd, and 5th sib-pairs, which need further exploration.

### Protein-protein interaction (PPI) network analysis of DEGs

Given that a fairly proportion of DEGs in each sib-pair are unique and that there might some similarities in gene expression pattern in MDD individuals, specifically in the 3rd, 4th, and 6th sib-pairs, we further tried to explore potential connections of the DEGs by protein-protein interaction network analysis (PPI). We firstly analyzed the PPI network of DEGs in each sib-pair separately to detect the potential MDD potential network at each case (Fig. S1, S2). We found that both the 1st and 5th sib-pairs had one dominant local network where the gene coding Parathyroid Hormone (PTH) was the hub (Fig. S1). In the 2nd sib-pair, *PTH* was also among the DEGs whereas no connection was detected, implying a somewhat weak of PTH network compared with 1st and 5th sib-pairs. Factually DEGs in the 2nd sib-pair had nearly no connection at all (Fig. S1). As to the 3rd, 4th and 6th sib-pairs that have more common features (Fig. 1), we found they had a fairly similar dominant network which also contains PTH (Fig. S2). These results suggest that despite genetic heterogeneity, *PTH* might be a potential gene associated with MDD, in the test samples. It is notable that in the dominant network generally shared in the 3rd, 4th, and 6th sib-pairs, there is another hub gene, i.e., fibroblast growth factor 2 (*FGF2*), which had more connected genes compared with *PTH* (Fig. S2), although *PTH* was still in the important position within the network (Fig. S2).

**Fig. 2:**
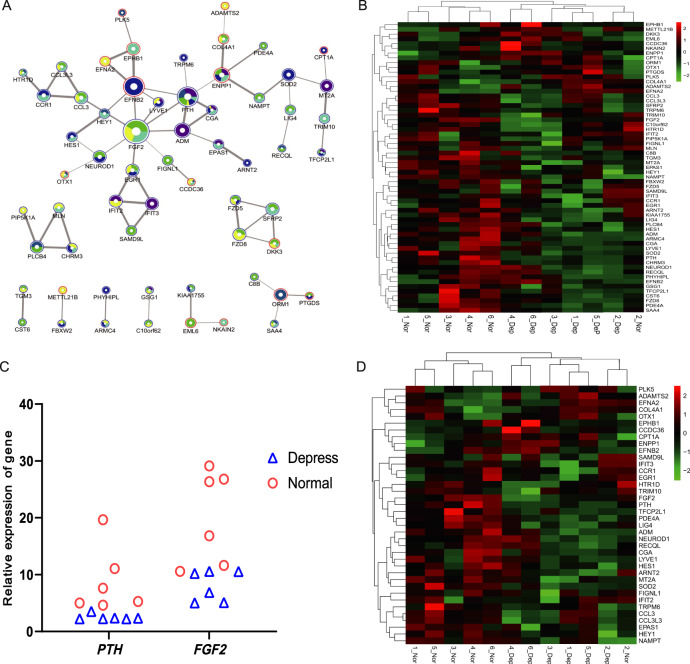
Protein interaction network and gene expression heatmap of the genes in the networks. **A** Protein-protein interaction networks constructed by the all DEGs in the six sib-pairs. Six colors are used to represent six pairs of siblings, purple for the first sib-pair, faint blue for the second sib-pair, green for the third sib-pair, yellow for the fourth sib-pair, dark blue for the fifth sib-pair, and lake blue for the sixth sib-pair. The red edge represents the up-regulated gene, and the blue edge represents the down-regulated gene. Hub gene are *FGF2* and *PTH*. **B** Expression heatmap and clustering of the genes in A. **C** Realtive expression of *PTH* and *FGF2* in six sib-pairs. *PTH* is a significantly differentially expressed gene shared by six sib-pairs, while *FGF2* only has a significant difference in 346 sib-pairs. **D** Expression heatmap and clustering of the genes in the subnetwork of PTH-FGF2 in A.

To further decipher the interaction of the genes that may share in all the tested samples, we included all the DEGs from each sib-pair and generated an integrated PPI (Fig. 2A). As expected, there was a remarkable dominant network bearing 38 genes, where *FGF2*, *PTH* were the outstanding hub genes. *FGF2* was shared in three sib-pairs and had 8 connections while *PTH* was shared in all the six tested sib-paired and had 6 connections (Fig. 2A). In addition, there were other eight small networks bearing two to four genes (Fig. 2A). It is notable that the two hub genes were generally down-regulated in MDD patients (Fig. 2B). Factually, the majority of DEGs in networks (45 out of 61) were generally down-regulated in MDD patients (Fig. 2A). These results implied reduced functional activity of these networks that might be influenced by MDD. We further investigated the expression pattern of these genes by heatmap and hierarchical clustering and found a relatively clearer divergence between the MDD patients and the normal sibs compared with the clustering generated by including all DEGs identified in each sib-pair (Fig. 1A), except for the 2nd sib-pair, in which the two sibs were still clustered together(Fig. 2C). Even when selecting DEGs in the dominant FGF2-PTH network, we still detected the same pattern (Fig. 2D). We checked the clinical characteristics of this pair and found that they were the only pair with two teenagers and the normal individual was only twelve. Maybe this is one potential factor that made them distinctive to other pairs in terms of gene expression.

### Functional enrichment analysis

**Fig. 3:**
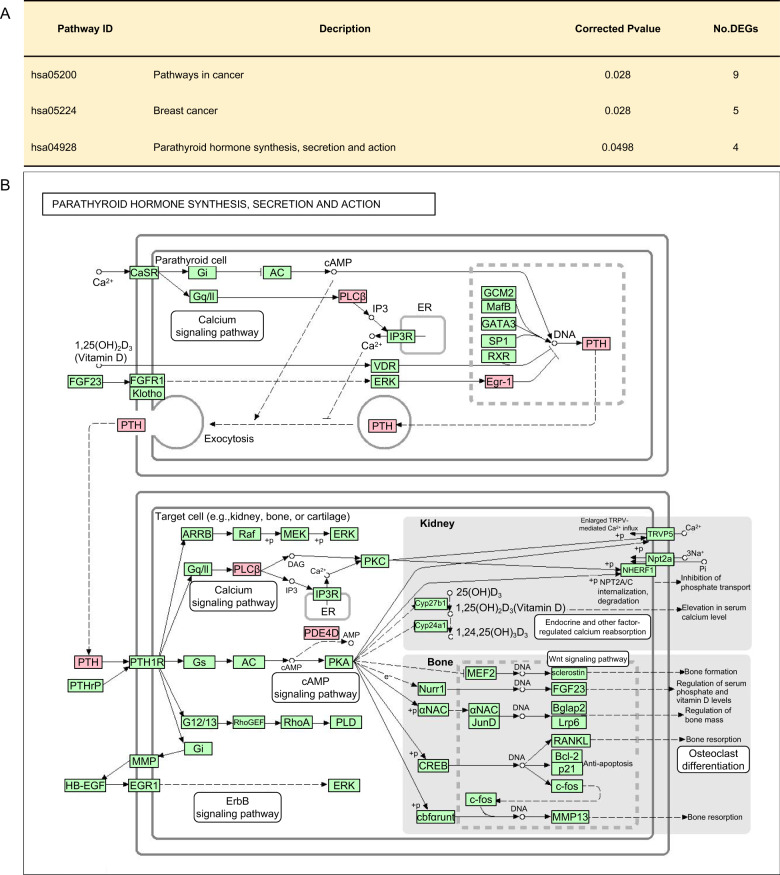
KEGG enrichments of DEGs in protein-protein interaction network (PPI). **A** There are three significant pathways, which are pathways in cancer, breast cancer, and parathyroid hormone synthesis, secretion, and action. **B** Schematic diagram of parathyroid hormone synthesis, secretion, and action. The genes in the pathway are labeled in red, which are *PTH*, *PLCB4*, *EGR1,* and *PDE4A*.

**Fig. 4:**
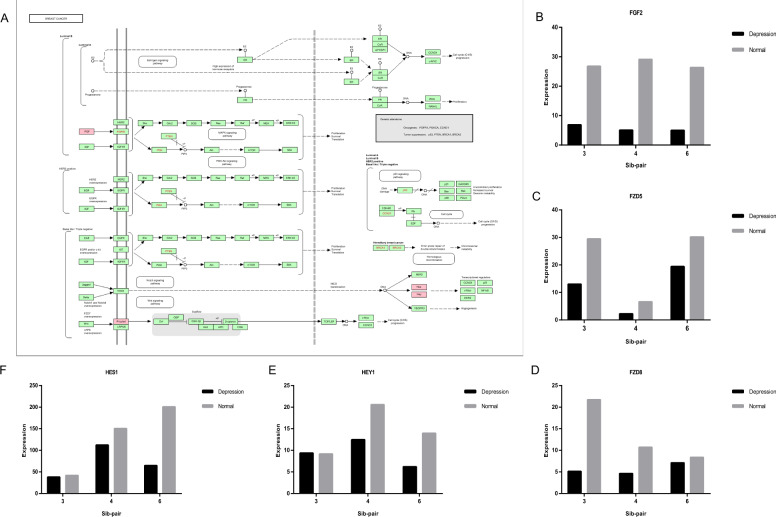
Breast cancer pathway and the expression values of differentially expressed genes (DEGs) in the pathway in third, fourth and sixth sib-pair. **A** Schematic diagram of the breast cancer pathway. The red rectangle markers indicate the family of DEGs in the pathway, which are *FGF2*, *FZD5*, *FZD8*, *HEY1,* and *HES1*. **B** The expression of *FGF2* gene in the third, fourth, and sixth sib-pair. **C** The expression of the *FZD5* gene in the third, fourth, and sixth sib-pair. **D** The expression of *FZD8* gene in the third, fourth, and sixth sib-pair. **E** The expression of *HEY1* gene in the third, fourth, and sixth sib-pair. **F** The expression of the *HES1* gene in the third, fourth, and sixth sib-pair. Black denotes depressed individual of sib-pair and gray indicates normal individual. The x-axis represents the sib-pair number and the y-axis represents the expression values of the gene.

To explore the functional implications of the above networks, we further generated GO and KEGG enrichment analyses on the differentially expressed genes identified in the protein-protein interaction networks. There were no significant enriched GO terms (data not shown). The result of KEGG enrichment analysis showed that there were three significant enriched pathways, i.e., two were related to cancer, i.e. pathway in cancer and breast cancer, and the other was the pathway of parathyroid hormone synthesis, secretion, and action (Fig. 3A). We firstly took breast cancer for example to decipher the detailed information of the DEGs in this pathway and found that they were mainly in the network of the 3rd, 4th, and 6th sib-pairs (Fig. 4). The hub gene *FGF2* was in this pathway. We found that these genes are involved in processes such as cell proliferation, organogenesis, and cancer-related transcriptional regulation. The five genes in this pathway were down-regulated in the peripheral blood lymphocytes of the MDD patients (Fig. 4), suggesting that the proliferation activity of these immune-related cells were reduced in the MDD patients of the three sib-pairs.

The pathway of parathyroid hormone synthesis, secretion, and action is factually the hub gene *PTH-related* pathway (Fig. 3B). The four genes *PTH*, *PLCB4*, *EGR1,* and *PDE4A* located in important nodes of this pathway and they were all down-regulated (Fig. 3B). Hence Parathyroid hormone was reduced in the blood in the MDD patients. Furthermore, we noticed that two genes, *PLCB4* and *PDE4A* that are important in downstream signaling pathways such as in calcium signaling and the second messenger cAMP pathway, respectively were also repressed, which influence bone and kidney function (Fig. 3B). These results indicate that the abnormal mental state of major depressive disorder may have a potential impact on physiological functions.

## Discussion

In this study, we generated comparative transcriptome analysis of peripheral blood lymphocytes from sib-pairs discordant of MDD and provided preliminary molecular cues of MDD in Chinese people. To our knowledge, this study is the first gene expression profile analysis of MDD using peripheral blood lymphocytes based on Chinese sib-pairs. Although the sample size is still small, the findings and implication in this study is valuable for further deep verification, which may be helpful for assistant diagnosis. Using these small samples, we were able to identify differentially expressed genes in each sib-pair (Fig. 1). We found fairly difference in terms of DGEs, suggesting possible genetic heterogeneity among sib-pairs. However, there were also some DGEs shared in certain sib-pairs. Further principle component and clustering analyses consistently reflected this pattern.

Some of the DGEs identified in our study were reported to show genetic variation in previous studies. A genome-wide association meta-analysis based in 135,458 cases and 344,901 controls was published in 2018 and identified 44 risk variants, of which the SNP, rs4143229, the gene nearest to that is *LACC1*, which was also found in our results [7]. Another quantitative review of whole-genome transcriptional data from 10 case-control studies reported the differentially expressed genes overlapped with our DEGs list, including *ADM*, *IFIT3*, *METTL21B*, *ADAMTS2*, *MCTP1,* and *LOC100996385* [14]. Besides, in two case-control studies of blood microarrays in major depressive disorder, a total of 165 genes were differentially expressed in both studies with the concordant direction of fold change, with the ADM gene also present in our DEGs [17]. However, our list of differentially expressed genes is not identical to those found in a recent study of depressive-normal twins, in which 30 DEGs were identified [21]. While in another study used genome-wide microarray gene expression from the peripheral blood of the elderly western population [27], with genetically unrelated case and control objects, we found that there were seven genes, *PLEKHA1, RECQL, RAGAPA1, C1ORF86, XPR1, NAMPT, and MCTP1*, were also identified in at least one sib-pair of our study. Furthermore, there were efforts on exploring epigenetic differences of discordant monozygotic twins [28–30], or using sib-pairs samples to explore the relationship between proband and patients and the genetic factors of depression [31, 32]. Methylation studies found that several CPG sites across which depressive co-twins from the discordant pairs, respectively is *CCDC181*, *RAB37*, *LHFP*, *KCND2*, *NGLY*1, *NUDT16P*, *TMEM81A*, *GANC*, *GHSR*. These studies using sib-pairs identified the alpha-haptoglobin (alpha-Hp) and third complement component (C3) loci and implied genetic linkage in depression spectrum disease. However, due to the heterogeneity of the MDD phenotype and all kinds of confounding differences between studies (such as race, type of participants, age, tissue/cell type, use of antidepressants, and other factors), there is no comparability in the results of comparing them under different circumstances, and the etiology of MDD is not determined by one gene or multiple genes. It is caused by many factors, which needs further in-depth exploration.

Although pending further validation in larger Chinese samples, here our pilot study highlighted two important cues that might be possibly associated with MDD. The first is parathyroid hormone (PTH) and it involved a pathway of parathyroid hormone synthesis, secretion, and action which were generally repressed in MDD individuals. The normal level of calcium and vitamin D in the human body is maintained by parathyroid hormone (PTH). The abnormality of calcium and vitamin D is directly or indirectly related to psychiatric features such as delusion, schizophrenia, cognitive impairment, mental illness, coma, mania, and various depressions [33]. In patients with depression, the conversion of tryptophan in the brain stops due to disregulation of parathyroid hormone, which produces little or no serotonin, further contributes to a defective mental state, changed cognition, and false sensory gait [33]. Defects in the processing of PTH may lead to hypoparathyroidism, resulting in hypocalcemia and numbness, which can cause psychiatric disorders. In an elderly patient with a long history of depression who developed chronic hypoparathyroidism after parathyroid adenoma surgery, when he was treated with calcium supplementation to restore serum calcium homeostasis, depression was completely eliminated [34]. These studies strongly supported that chronic hypoparathyroidism marked by a reduced level of PTH, may be a correlative factor in the development of depression.

It is notable that in western human samples, there are still controversial results in terms of the association of PTH and MDD. A study report there were no associations between serum concentrations of 25-hydroxyvitamin D and parathyroid hormone and depression among US adults based on a cross-sectional, population-based sample (including 3916 participants aged ≥ 20 years) from the 2005-6 National Health and Nutrition Examination Survey [35]. Results from a national population-based household sample of 4,002 Jordanian participants aged ≥ 25 years exhibited that no significant association was found between serum PTH levels and depression [36]. Furthermore, these studies tested the content of serum PTH instead of lymphocyte. Jamilian compared serum levels of vitamin D, calcium, phosphorus and parathyroid hormone in depressed patients and healthy subjects in an Iranian population and discovered vitamin D and parathyroid hormone level in healthy participants was significantly higher than depressed patients [37] In order to evaluate possible pathogenic mechanisms implicated the association of vitamin D status with major depressive disorder, it has been conducted with Spanish [38], Jordanians [36], Malaysian women [39] and Chinese elderly people [40], measuring serum parathyroid hormone level to reflect from the side whether there is a correlation between vitamin D and depression. Here our study provided cues of reduced expression of PTH at the transcriptional level, in peripheral blood lymphocytes of MDD individuals. Further investigation in more Chinese samples will help clarify this insight.

The other interesting finding is the fibroblast growth factor (FGF2) and its involved pathway of cancer, specifically breast cancer which were generally repressed in MDD individuals of 3 out of 6 sib-pairs, indicating an additional potential association with MDD occurred in some Chinese MDD patients. FGF2 belongs to the FGF family binding to heparin and have a wide range of mitogenic and angiogenic activities. This protein is involved in a variety of biological processes, such as limb and nervous system development, wound healing, and tumor growth.

The fibroblast growth factor FGF2 is one of the major neurotrophic proteins and plays an important role in the central nervous system (CNS). Hence the role of FGF2 and related networks in depression are well documented in the brain [41]. There is growing evidence that the expression of the *FGF2* gene is down-regulated in the brain region of depressed patients and plays an antidepressant role in animal models of depression [41–44]. In another case, FGF2 was reported to decrease the expression of CTGF, a possible pre-depression molecule, in the adult dentate gyrus [45]. It was also reported that genes positively regulated by *FGF2* included *EGR1*, *Etv4*, *SPRY4,* and *DUSP6* in non-neuronal cell types [42].

Here in our study, we detected generally repressed expression of *FGF2* and its related genes enriched in the breast cancer pathway, including *EGR1*, in peripheral blood lymphocytes of certain MDD individuals. A recent study measured serum FGF2 levels in the 28 MDD patients before and after treatment and 30 healthy controls using enzyme-linked immunosorbent assay, and found that serum FGF2 levels in patients with depression were significantly lower than those in healthy controls [46], consistent to our result. We suspected that the result might suggest a reduced proliferative potential of these lymphocytes, although the MDD individuals also have normal values in whole blood cell analyses (data not shown). The low proliferative potential of lymphocytes may cause a decrease in immunity, brought an additional healthy risk of some MDD patients. Pending on large sample validation, *FGF2,* and related pathway may become a potential molecular targets to an exploration of the pathogenesis of MDD, and provide new ideas for the diagnosis and treatment of depression in the future.

Despite the above two interesting findings, the main limitation of this study is the small sample size. We anticipate that a large sample size of well-matched psychiatric controlled samples (monozygotic twins discordant on MDD without medication and suicide) should help to analyze the effects of depression in future studies. Secondly, the age span of our sample is relatively large, particularly including teenagers. In our study, we are not able to distinguish the MDD from the normal one in this teenager sib-pair, using the microarray data. Maybe there is still a novel molecular mechanism related to MDD in teenagers, which should be paid more attention in the near future.

## Supplementary information

supplmentary figure file

## Data Availability

The gene expression profile data in this study can be requested from the corresponding author according to the needs.
