# Correction to: Gene expression profiling in peripheral blood lymphocytes for major depression: preliminary cues from Chinese discordant sib-pair study

**DOI:** 10.1038/s41398-021-01726-8

**Published:** 2021-11-29

**Authors:** Chan Wu, Danfeng Wang, Kangkang Niu, Qili Feng, Hanwei Chen, Haibing Zhu, Hui Xiang

**Affiliations:** 1 South China Normal University-Panyu Central Hospital Joint Laboratory of Basic and Translational Medical Research, Guangzhou Panyu Central Hospital, Guangzhou, China; 2 Guangdong Provincial Key Laboratory of Insect Developmental Biology and Applied Technology, Guangzhou Key Laboratory of Insect Development Regulation and Application Research, Institute of Insect Science and Technology, School of Life Sciences, South China Normal University, Guangzhou, 510631 China; 3 The Affiliated Brain Hospital of Guangzhou Medical University, Guangzhou, China; 4 Department of Psychiatry, Guangzhou Panyu Central Hospital, Guangzhou, China

**Keywords:** Clinical genetics, Comparative genomics, Predictive markers, Depression

Correction to: *Translational Psychiatry* 10.1038/s41398-021-01665-4, published online 19 October 2021

The original version of this article unfortunately contained errors in the affiliations. The South China Normal University-Panyu Central Hospital Joint Laboratory of Basic and Translational Medical Research actually belongs to the Guangzhou Panyu Central Hospital. Therefore, “Guangzhou Panyu Central Hospital” should be added to this affiliation. The corresponding author Hui Xiang is also a professor in the Joint Laboratory and in this study, and Hui Xiang is on duty as the professor in the Joint Laboratory. Therefore, the first affiliation should be added with it.

