# Supplementary material for: Gene expression profiling in peripheral blood lymphocytes for major depression: preliminary cues from Chinese discordant sib-pair study
Source: Transl Psychiatry. 2021 Oct 19;11:540. doi: 10.1038/s41398-021-01665-4 (PMC8526709; doi:10.1038/s41398-021-01665-4)
Supplement: Supplementary file 1 — supplmentary figure file [file 41398_2021_1665_MOESM1_ESM.pdf]

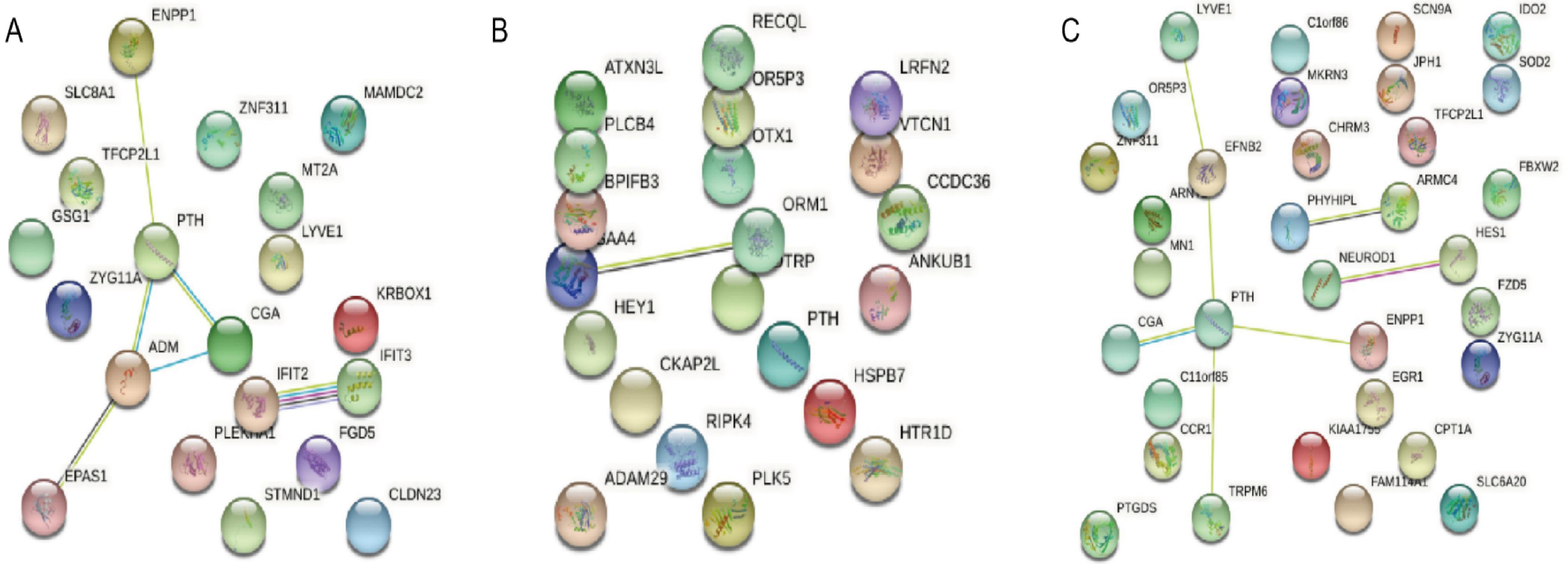

Fig. S1 Protein-protein interaction network (PPI) of the first, second and fifth sib-pair respectively. **A** PPI of the first sib-pair. **B** PPI of the second sib-pair. **C** PPI of the fifth sib-pair.

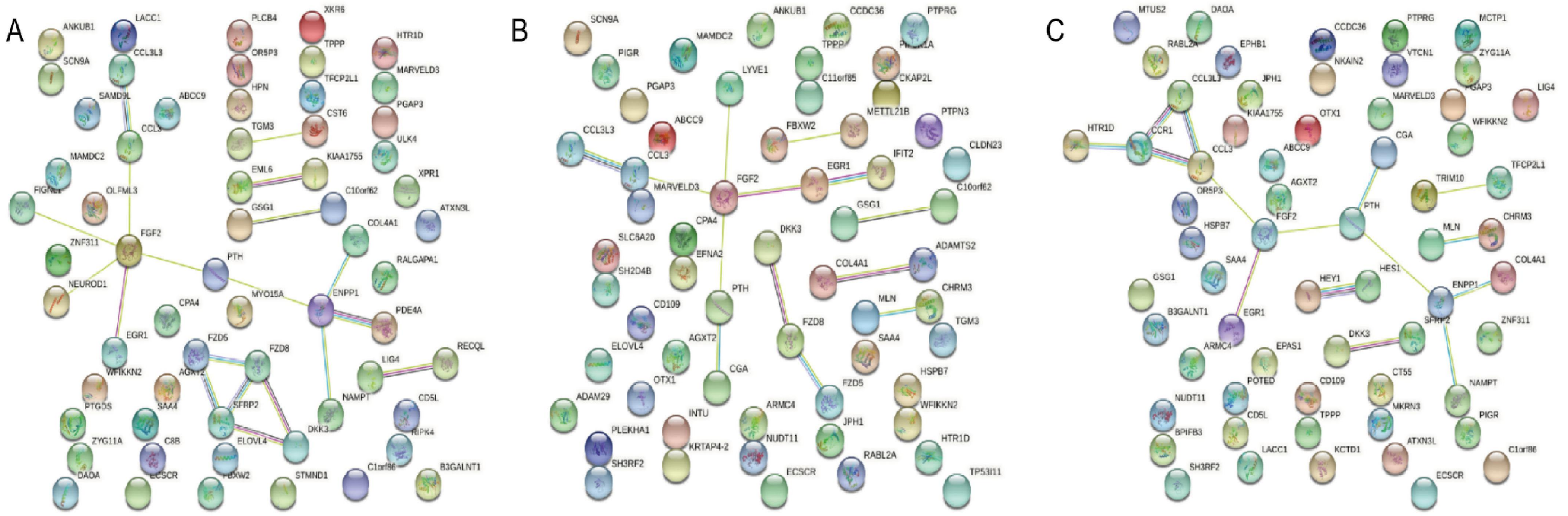

Fig. S2 Protein-protein interaction network (PPI) of the third, fourth and sixth sib-pair respectively. A. PPI of the third sib-pair. **B.** PPI of the fourth sib-pairs. **C.** PPI of the sixth sib-pair.
